# Long-Term Outcome of Enterprise Stenting for Symptomatic ICAS in a High-Volume Stroke Center

**DOI:** 10.3389/fneur.2021.672662

**Published:** 2021-06-17

**Authors:** Rongrong Cui, Long Yan, Kaijiang Kang, Ming Yang, Ying Yu, Dapeng Mo, Feng Gao, Yongjun Wang, Xin Lou, Zhongrong Miao, Ning Ma

**Affiliations:** ^1^Interventional Neuroradiology, Beijing Tiantan Hospital, Capital Medical University, Beijing, China; ^2^China National Clinical Research Center for Neurological Diseases, Beijing, China; ^3^Center of Stroke, Beijing Institute for Brain Disorders, Beijing, China; ^4^Department of Neurology, Beijing Tiantan Hospital, Capital Medical University, Beijing, China; ^5^Department of Radiology, Chinese People's Liberation Army (PLA) General Hospital, Beijing, China

**Keywords:** intracranial atherosclerotic stenosis, ICAs, endovascular treatment, enterprise stent, long-term outcomes

## Abstract

**Background and Purpose:** The Enterprise stent has been used for treating intracranial atherosclerotic stenosis (ICAS), but its long-term outcome remains unclear. The purpose of this study was to evaluate the long-term clinical efficacy of the Enterprise stent used for patients with symptomatic ICAS due to hypoperfusion.

**Method:** Patients with symptomatic ICAS due to hypoperfusion treated with the Enterprise stents from a high-volume stroke center were evaluated. The successful recanalization was defined as the Modified Thrombolysis In Cerebral Infarction (mTICI) ≥ 2b. The stroke and neurological death that occurred within 72 h after the procedure as well as long-term clinical and imaging outcomes were analyzed.

**Results:** Overall, 130 patients with 130 ICAS treated with the Enterprise stent were included in our study. The successful recanalization rate was 100%. The mean pre- and postprocedural stenosis was 82.9 ± 8.9% vs. 15.1 ± 8.4%. Periprocedural complications occurred in 5 (3.8%) patients within 72 h after the procedure. Clinical follow-up data were available in 125 (96.2%) patients (median, 24 months) and any stroke or neurological death was encountered in 6 (4.8%) patients. Angiographic follow-up data was obtained from 118 (90.8%) patients (median, 13.5 months). In addition, 1-year in-stent restenosis (>70%) was found in 17 (14.4%) patients, and among them, 4 (23.5%) patients were symptomatic.

**Conclusion:** Deployment of Enterprise stent is safe for ICAS. The short-term and long-term outcomes were acceptable, but the efficacy of the Enterprise stent needs to be further evaluated in future studies.

## Introduction

Intracranial atherosclerotic stenosis (ICAS) is one of the most important causes of ischemic stroke, especially in Asian populations, which is responsible for 33–56% of strokes in Asia (1–4). So far, dual antiplatelet combined with control of risk factors is the first-line treatment for patients with symptomatic ICAS. However, the SAMMPRIS trial and the VISSIT trial revealed a high 1-year stroke recurrence (12.2 and 15.1%) under medical therapy (5, 6), suggesting aggressive medical treatment was not enough to achieve the desired preventive effect.

The WEAVE/WOVEN trial demonstrated a low rate of periprocedural complication (2.6%) and stroke recurrence (8.5%) in patients with ICAS treated with Gateway balloon dilation plus Wingspan stent implantation (Stryker, Kalamazoo, MI) by experienced interventionalists (7, 8).

The Wingspan stent was loaded with an over-the-wire delivery system with a dual tapered tip. These features make it difficult in being positioned in stenosis with distal artery with a smaller caliber or lesions with tortuous access proximally (9–11). In China, a balloon-mounted stent (Apollo, MicroPort Neuro Tech, Shanghai, China) has been used to treat ICAS, but the stiffness of the delivery system made the stent difficult through the lesion when treating ICAS with tortuous access (12).

Recently, there are some studies exploring stents for assisting coiling for the treatment of wide-necked intracranial aneurysms in treating ICAS as off-label use. The Enterprise stent with closed-cell design (Codman Neurovascular, Raynham, Massachusetts, USA) is one of them. On account of its advancement and release by a catheter system, the device may be used as an alternative to the wingspan system for treating ICAS. So far, several studies had reported the efficacies of the Enterprise stenting for ICAS, but few studies have focused on the long-term outcome in a high-volume stroke center (11, 13–19).

We conducted the study to evaluate the long-term clinical efficacy of the Enterprise stent for symptomatic ICAS.

## Methods

### Study Design and Population

This study was a retrospective study, which had been approved by the institutional review board. Written informed consent was obtained from the patients or their legally authorized representatives.

We retrospectively identified individuals with symptomatic ICAS, which had been treated with the Enterprise stents from databases in a high-volume stroke center. The inclusion criteria included patients between 18 and 80 years old with the transient ischemic attack (TIA) or ischemic stroke due to ICAS (70–99%), located at the intracranial internal carotid artery (ICA), the intracranial vertebral artery (VA), the M1 segment of the middle cerebral artery (MCA), or the basilar artery (BA), and stented with Enterprise ≥8 days after their last ischemic event. TIA is defined as a transient episode of neurologic dysfunction characterized by a complete and spontaneous clinical resolution of symptoms within 24 h, without acute infarction showed on the brain MR and no permanent neurological deficit (20). The symptoms were related to hypoperfusion in the territory of the target artery with cerebral blood flow (CBF) decrease of ≥30% compared with the contralateral side on computer tomography perfusion (CTP) or the American Society of Interventional and Therapeutic Neuroradiology/Society of Interventional Radiology (ASITN/SIR) scale ≤ 2 on digital subtraction angiography (DSA) (21). The definition of the stenotic degree was the same as the WASID method (22). The exclusion criteria included (1) non-atherosclerotic stenosis (such as dissection, vasculitis, or fibromuscular dysplasia), (2) tandem stenosis, (3) target lesion planted with any other stent previously, (4) concurrent aneurysm, intracranial space-occupying lesions, and arteriovenous malformations, and (5) total occlusion lesions.

### Procedure

The procedures were performed by experienced neurointerventionalists (performing more than 100 intracranial endovascular procedures per year). After general anesthesia and systemic heparinization, a 6F or 5F Envoy guiding catheter (Cordis Neurovascular, USA), or a long sheath combined with an intermediate catheter, was placed into the proximal segment of the target lesion. Based on morphology, target lesions were classified as Mori type A, type B, or type C (23). Under a roadmap, a microwire without or with a microcatheter crossed the lesion to the distal true lumen. Pre-dilation with a balloon that matches the diameter of the lesion was performed. The diameter of the balloon selected was 80–90% of the diameter of the reference artery (60–80% when the stenosis adjacent to angiographically visible perforators). After retrieving the balloon catheter, a Prowler Select Plus microcatheter (Codman, Raynham, Massachusetts, USA) was placed at the normal vessel distal to the lesion. The length of the Enterprise stent was longer than that of the lesion. If stent positioning is satisfactory, retract the microcatheter to allow the stent to deploy across the whole lesion. A post-dilation was performed if residual stenosis (≥50%) was observed. Successful recanalization was defined as the Modified Thrombolysis In Cerebral Infarction (mTICI) ≥ 2b (24).

### Periprocedural Management

All patients were given a dual antiplatelet regimen (aspirin 100 mg and clopidogrel 75 mg daily) for at least 5 days before the procedure. They were maintained on aspirin (100 mg/d) plus clopidogrel (75 mg/d) for 90 days after stenting. If the patients had a low activity to aspirin or clopidogrel suggested by the Thromboelastography (TEG) (inhibition rate of arachidonic acid <50% or adenosine diphosphate <30%), ticagrelor or cilostazol was chosen as an alternative after consulting vascular neurologists. All patients were given a weight-based dose of 0.4–0.6 mL nadroparin (Fraxiparine; Sanofi Winthrop Industry) every 12 h for 1–2 days after intracranial hemorrhage was excluded by post-procedure computer tomography (CT). Strict blood pressure management was performed from the start of the procedure to 72 h after the procedure to prevent hyperperfusion syndrome (the systolic blood pressure was around 100 mmHg during the procedure and around 120 mmHg after the procedure).

### Clinical and Imaging Follow-Up and Study Outcomes

**Figure 1:**
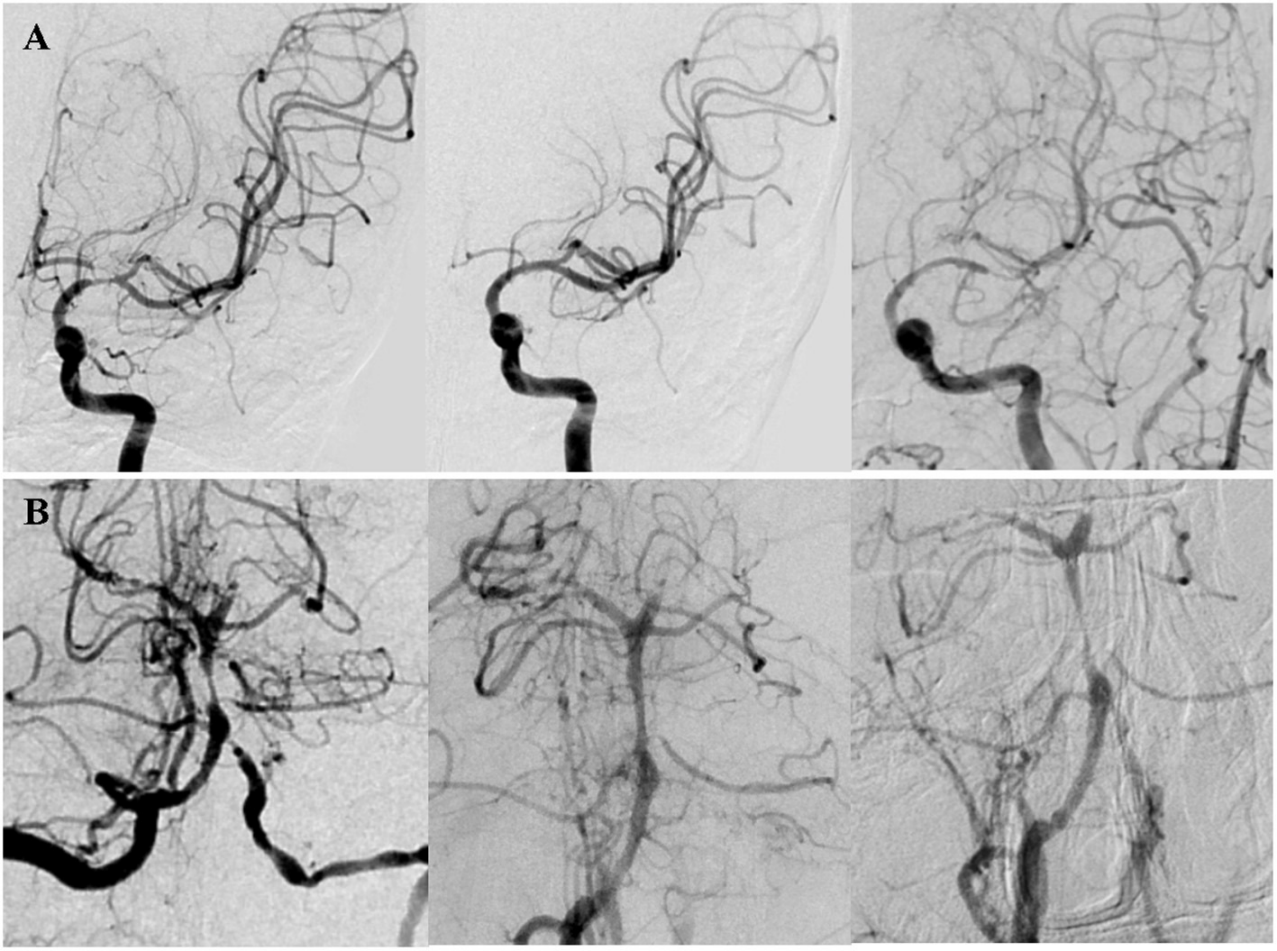
Two patients with in-stent restenosis (ISR) by DSA. **(A)** A patient presented with right limb weakness and numbness for 10 months due to severe stenosis (70%) at the M1 segment of the left MCA. The Mori classification was type C. The lesion was dilated with a 2.25 × 9 mm Gateway balloon (Boston Scientific, Fremont, CA) and implanted a 4 × 23 mm Enterprise stent (Codman Neurovascular, Raynham, Mass). Postoperative DSA showed the degree of stenosis decreased to 5%. DSA after 17 months of the procedure showed significant in-stent restenosis (ISR). **(B)** A patient presented with bilateral lower limbs weakness and diplopia for 2 months due to high-grade stenosis (80%) at the middle segment of the BA. The lesion was dilated with a 2 × 9 mm Gateway balloon and implanted a 4.5 × 14 mm Enterprise stent. Postoperative DSA showed the degree of stenosis decreased to 35%. DSA after 6.33 months of the procedure showed significant ISR.

**Figure 2:**
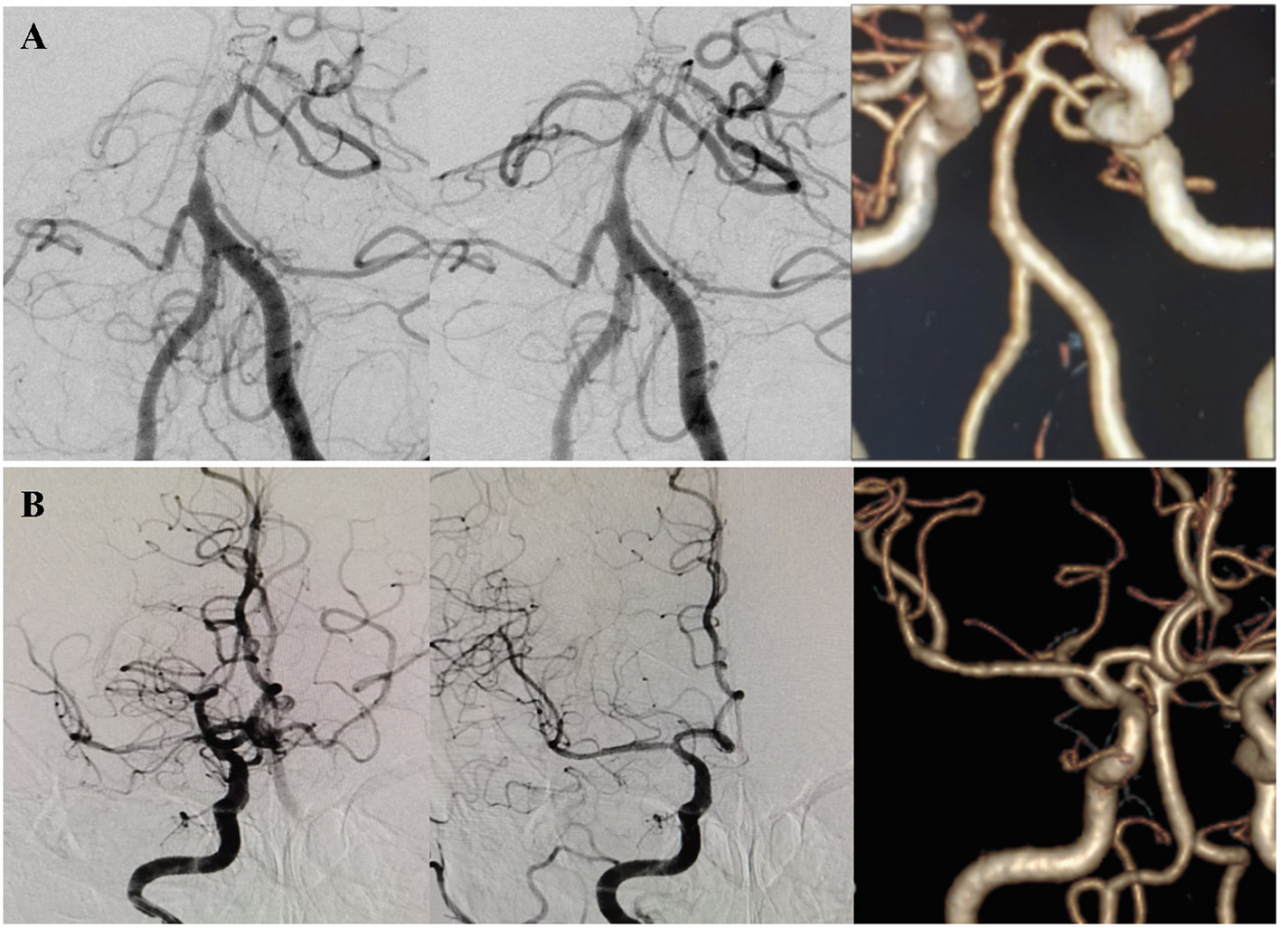
Two patients with no significant ISR by CTA. **(A)** A patient presented with dizziness and diplopia for 2 months. DSA showed a high-grade (>90%) stenosis at the middle segment of the basilar artery, classified as Mori B type. The lesion was dilated with a 2 × 9 mm Gateway balloon and implanted a 4.5 × 14 mm Enterprise stent. Postoperative DSA showed the degree of stenosis decreased to 30%. CTA after 54.5 months of the procedure showed no significant ISR. **(B)** A patient presented with left limb weakness and dysarthria for 10 days due to a high-grade (>90%) stenosis at the M1 segment of the right MCA. The Mori classification was type C. The lesion was dilated with a 2 × 9 mm Gateway balloon and implanted a 4.5 × 22 mm Enterprise stent. Postoperative DSA showed the degree of stenosis decreased to 5%. CTA after 23 months of the procedure showed no significant ISR.

Clinical and imaging follow-up was collected and reviewed by trained personnel. All patients received clinic visits, social software, or telephone interviews by trained personnel. The imaging data were interpreted by two neuroradiologists. The neurological function was evaluated by modified Rankin Scale (mRS) scores. For patients with new neurological symptoms, magnetic resonance imaging (MRI) or computer tomography angiography (CTA) was obtained. For patients evaluated with DSA, in-stent restenosis (ISR) was defined as a >70% narrowing of the stented segment or immediately adjacent (within 5 mm) to the implanted stent (Figure 1). For patients evaluated with CTA, the stents were considered as ISR if the stented segment or the proximal and distal parent vessel could not be well-visualized or showed an apparent filling defect (Figure 2). In cases evaluated with magnetic resonance angiography (MRA) in which the findings on the cross-sectional imaging of 3D time-of-flight (TOF) were ambiguous or suggestive of ISR, DSA was performed (25). In one case with ISR, only MRA was available as a follow-up examination. For patients who performed follow-up DSA and CTA simultaneously, the degree of ISR was measured and compared for evaluating the consistency between these two imaging modalities. For patients evaluated with transcranial doppler ultrasonography (TCD), if the TCD data demonstrated abnormal flow velocity and direction of stented arteries, CTA, or DSA was required.

The primary outcome was any stroke (including ischemic or hemorrhagic stroke) and neurological death within 72 h after the procedure, and that during the follow-up period. The definitions of the study outcomes were the same as they were defined in the WEAVE/WOVEN trial.

The secondary outcome was composed of the assessment of the degree of neurological deficit in stroke patients and the ISR. We classified stroke into minor and major types according to the National Institutes of Health Stroke Scale (NIHSS) score (worsening of the NIHSS score ≤ 3 as minor stroke, >3 as major stroke).

### Statistical Analysis

Continuous variables were expressed as mean ± SD for normally distributed data or as median with an interquartile range (IQR) for data with skewed distribution, while categorical variables are presented as numbers and percentages. Cohen kappa statistic was used to compare the consistency of the degree of ISR measured by CTA and DSA. The χ2 test was used to analyze differences between the present study and the WEAVE/WOVEN study and differences in 1-year ISR rates among patients with different Mori types of the lesion and between patients with lesions located at different arteries. A *P*-value < 0.05 was considered a statistically significant difference. The statistical analysis was performed using a commercial SPSS statistical software package.

## Results

### Baseline Features of Enrolled Patient

**Figure 3:**
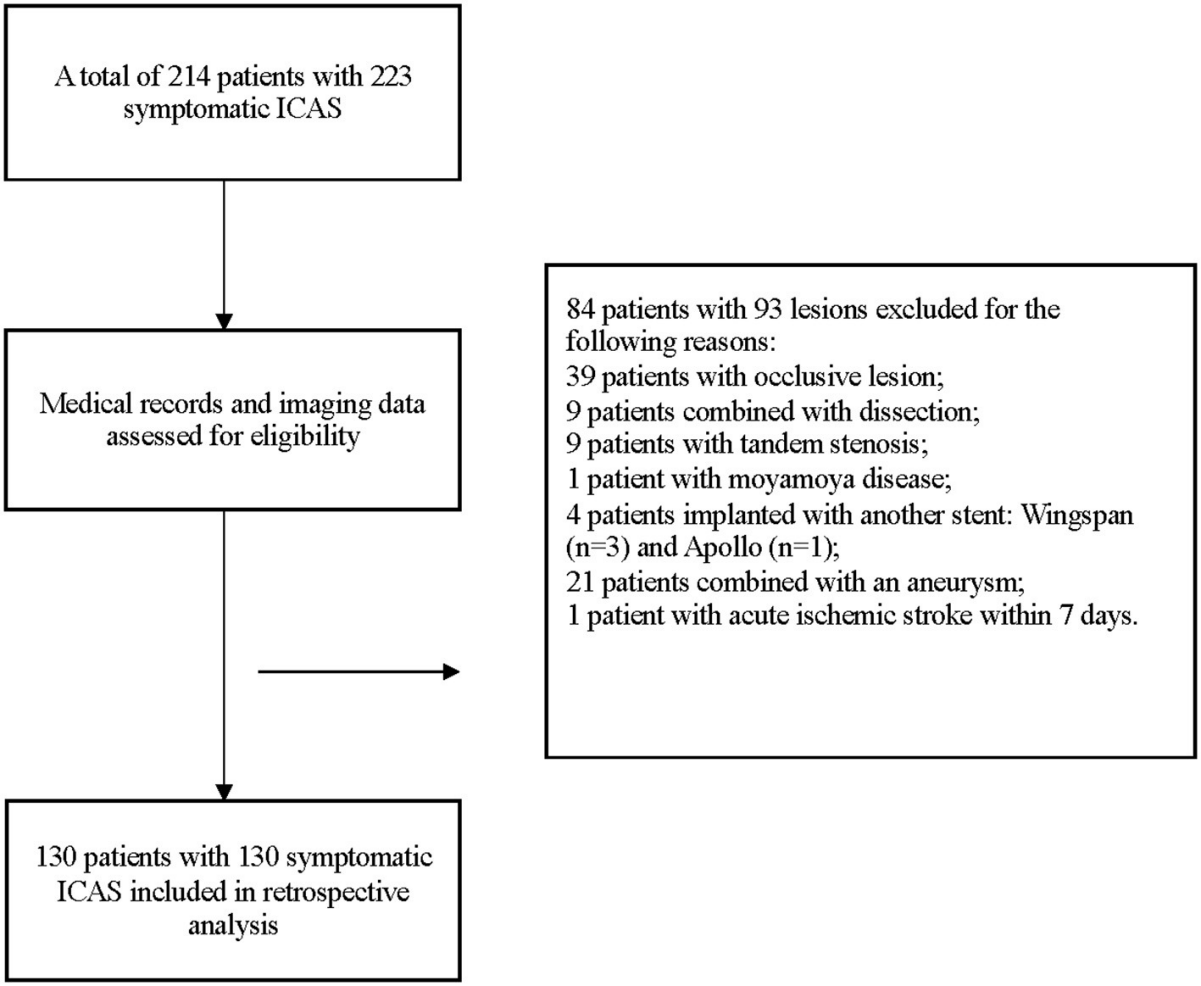
Selection of patients or lesions for analysis in the study.

From May 2015 to August 2019, a total of 214 patients with 223 symptomatic ICAS were treated with the Enterprise stent. Among them, 130 patients with 130 lesions treated with Enterprise stent were included eventually. The details of enrollment were displayed in Figure 3.

The mean age of patients was 59.2 ± 8.5 years, and 66.2% of patients were male. Of the 130 patients, 104 patients (80%) were complicated with hypertension, 60 (46.2%) with diabetes mellitus, and 42 (32.3%) with hyperlipidemia. The main qualifying event included stroke in 60% and TIA in 40% of the patients. The time from the latest ischemic event to stenting was 1 month (median). Most of the lesions were located at the MCA (31.5%, 41/130), BA (36.2%, 47/130), and VA (22.3%, 29/130). The baseline characteristics were summarized in Table 1.

**Table 1:** Comparison of baseline data between the WEAVE/WOVEN trial and the present study.

**Characteristic**	**Wingspan (WEAVE/WOVEN, *N* = 152)**	**Enterprise (present study, *N* = 130)**	***P*-value**
Age, mean ± SD	61.9 ± 10.5	59.2 ± 8.5	/
Male, *N* (%)	81 (53.3%)	86 (66.2%)	0.028
**Comorbidities**, ***N*** **(%)**			
Hypertension	140 (92.1%)	104 (80%)	0.003
Diabetes mellitus	91 (59.9%)	60 (46.2%)	0.021
Hyperlipidemia	131 (86.2%)	42 (32.3%)	<0.001
**Risk factors**, ***N*** **(%)**			
Smoker	80 (52.6%)	62 (47.7%)	0.408
Alcohol use	/	59 (45.4%)	/
**Ischemic events**, ***N*** **(%)**			
TIA	0 (0.0%)	51 (39.2%)	<0.001
Stroke	152 (100%)	79 (60.8%)	<0.001
**Possible cases with resistance by TEG**			
Arachidonic acid	/	6/109 (5.5%)	/
Adenosine diphosphate	/	43/109 (39.4%)	/
Time to stent from the latest ischemic event (days), median (IQR)	22	30 (30–60)	/
**Location**, ***N*** **(%)**			
Anterior circulation	102 (65%)	54 (41.5%)	<0.001
ICA	40 (25.5%)	13 (10%)	<0.001
MCA	62 (39.5%)	41 (31.5%)	0.108
Posterior circulation	55 (35%)	76 (58.5%)	<0.001
BA	22 (14.0%)	47 (36.2%)	<0.001
VA	32 (20.4%)	29 (22.3%)	0.799
PCA	1 (0.6%)	0 (0.0%)	0.354
**mRS score**, ***N*** **(%)**			
0	20 (13.2%)	90 (69.2%)	<0.001
1	37 (24.3%)	23 (17.7%)	0.174
2	52 (34.2%)	10 (7.7%)	<0.001
3	43 (28.3%)	4 (3.1%)	<0.001
4	0 (0.0%)	3 (2.3%)	0.193
5	0 (0.0%)	0 (0.0%)	/
Mori classifications, *N* (%)			/
Mori A	/	13 (10%)	
Mori B	/	65 (50%)	
Mori C	/	52 (40%)	
Percent (%) stenosis baseline			/
Mean ± SD	83.2 ± 8.3	82.9 ± 8.9	
Median (IQR)	82.0 (77.0–91.0)	85.0 (75.0–90.0)	
Range (min, max)	(40.0, 97.0)	(70.0, 99.0)	
Percent (%) stenosis after stenting			/
Mean ± SD	28.3 ± 16.9	15.1 ± 8.4	
Median (IQR)	27.5 (14.0–41.0)	10 (10–20)	
Range (min, max)	(0.0, 84.0)	(0, 40)	
Length of the target lesion (mm)			/
Mean ± SD	/	10.4 ± 5.1	
Median (IQR)	/	9.3 (6.9–13)	
Range (min, max)	/	(2.5, 30)	
Stent diameter (mm)			/
Mean ± SD	/	4.4 ± 0.2	
Median (IQR)	/	4.5 (4–4.5)	
Range (min, max)	/	(4, 4.5)	
Stent length (mm)			/
Mean ± SD	/	22.7 ± 6.0	
Median (IQR)	/	22 (22–23)	
Range (min, max)	/	(14, 39)	
Pre-stent TICI grade, *N* (%)			/
0	/	0 (0.0%)	
1	/	13 (10%)	
2a	/	22 (16.9%)	
2b	/	91 (70%)	
3	/	4 (3.1%)	

*TIA, transient ischemic attack; TEG, thromboelastography; BA, basilar artery; ICA, internal carotid artery; MCA, middle cerebral artery; PCA, posterior cerebral artery; VA, vertebral artery; mRS, modified Rankin Scale; mTICI, modified Thrombolysis In Cerebral Infarction*.

### Treatment Characteristics

Of the 130 patients with 130 ICAS, successful recanalization was acquired in all patients (100%). Among them, one patient was implanted with two stents due to a long lesion. In total, 13 lesions were classified as Mori A type (10%), 65 as Mori B type (50%), and 52 as Mori C type (40%). The mean rate of stenosis improved from 82.9 ± 8.9% to 15.1 ± 8.4% after the procedure. The treatment and technical characteristics were demonstrated in Table 1.

### Peri-Procedure Complications

In total, five patients (3.8%, 5/130) experienced complications within 72 h after the procedure, including one death attributable to hyper-perfusion intracranial hemorrhage after MCA stenting, one patient with major stroke due to a frontal lobe hemorrhage with subarachnoid hemorrhage (SAH) after MCA stenting, two patients with minor ischemic stroke due to in-stent thrombosis (one MCA stenting and the other VA stenting), and one patient with minor hemorrhagic stroke after VA stenting. The complication prevalence after MCA stenting was relatively higher than other locations but without significant difference (*p* = 0.215).

### Long-Term Outcomes

**Figure 4:**
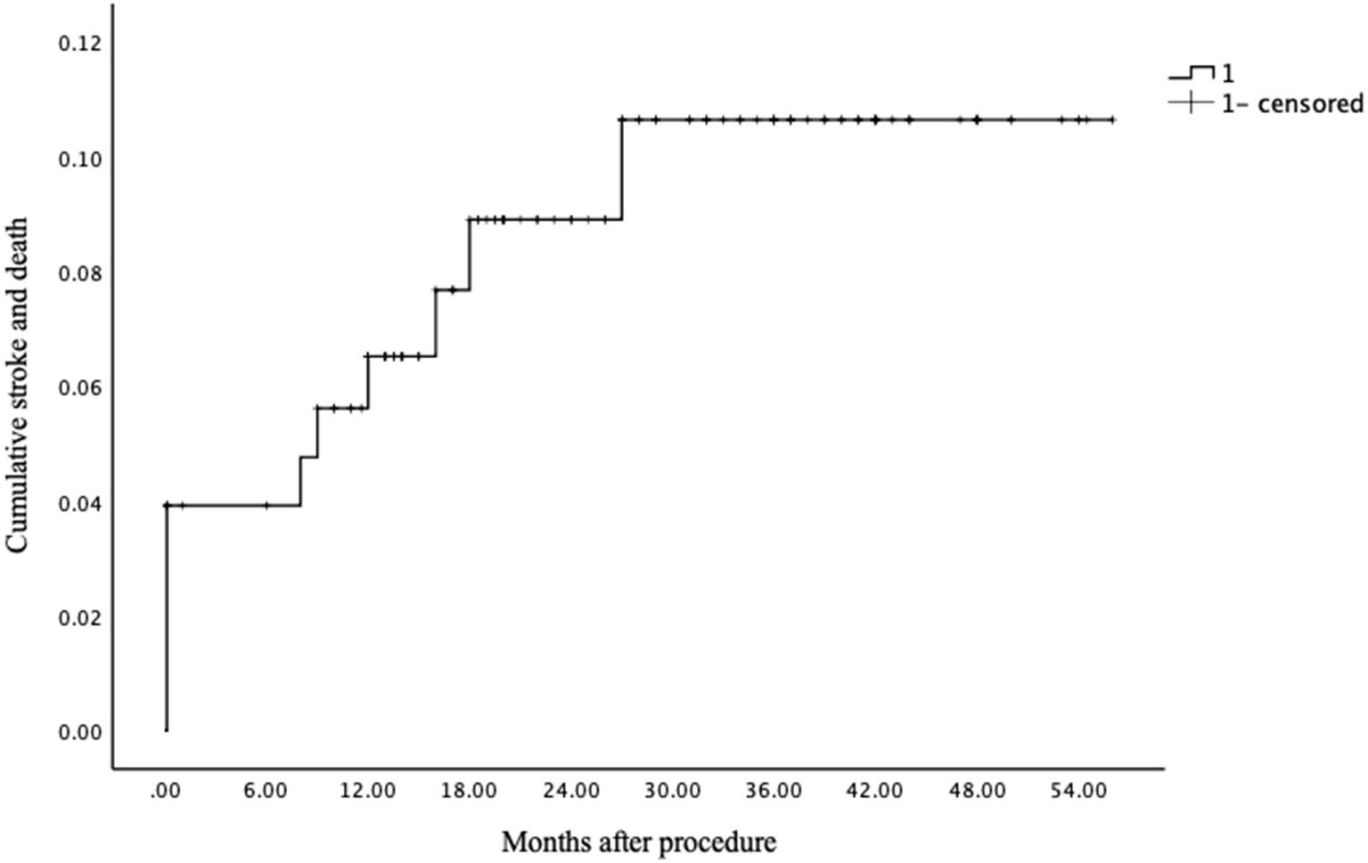
Kaplan-Meier curve of cumulative stroke and death rate of the included patients in the present study.

A total of 125 patients (96.2%) had clinical follow-up data (mean, 27.2 ± 13.5 months). Among them, the follow-up period of 124 patients (95.4%) was more than 6 months after the procedure, 121 patients (93.1%) more than 12 months, 90 patients (69.2%) more than 18 months, and 69 patients (53.1%) more than 24 months. After a mean follow-up period of 27.2 months, 102 (81.6%) patients achieved a score of 0, 8 (6.4%) patients a score of 1, 4 (3.2%) patients a score of 2, and 11 (8.8%) patients a score of 3–6 in mRS. Stroke or death occurred in six patients (4.8%), including one death, three major strokes, and two minor strokes (Table 2). Therefore, including 5 patients with periprocedural stroke and neurological death, 11 of the 125 patients (8.8%) had an index stroke or death within the clinical follow-up period (Figure 4).

**Table 2:** Stroke in the territory of the stented artery after 30 days.

**Number**	**Responsible artery**	**Months after procedure stroke/death occurred**	**ISR status**	**Modality**	**Death/major stroke/minor stroke**
1	BA	9	Yes	MRA	Death
2	MCA	8	Yes	CTA	Major Stroke
3	BA	16	No	DSA	Major Stroke
4	MCA	12.5	Yes	CTA	Major Stroke
5	MCA	12	Yes	DSA	Minor stroke
6	ICA	27	Yes	DSA	Minor stroke

*BA, basilar artery; ICA, internal carotid artery; MCA, middle cerebral artery; ISR, in-stent restenosis; DSA, digital subtraction angiography; CTA, computer tomography angiography; MRA, magnetic resonance angiography*.

### In-Stent Restenosis

During a mean time of 17.9 ± 11.3 months, 118 of 130 patients (90.8%) had available vascular follow-up data, including 88 patients (74.6%) with CTA, 17 (14.4%) with DSA, 3 (2.5%) with MRA, and 10 (8.5%) with TCD. Among them, the follow-up period of 113 patients (86.9%) was more than 6 months after the procedure, 94 patients (72.3%) more than 12 months, 50 patients (38.5%) more than 18 months. Among 118 patients, 11 patients were evaluated by CTA and DSA simultaneously, and the consistency of the degree of ISR measured by CTA and DSA was high (κ, 0.744; 95% CI: 0.281–1.207). ISR or in-stent occlusion within 1 year after the procedure was reached by 17 of 118 (14.4%) patients, and 9 (52.9%) of them were detected by DSA, 7 (41.2%) by CTA, and 1 (5.9%) by MRA at a mean time of 8.6 ± 3.3 months after the procedure. In total, 4 of 17 (23.5%) ISRs were symptomatic (Table 2). Stratification of the 1-year ISR according to the stented artery and Mori types showed there was no significant difference (Table 3). The details of the outcomes are given in Table 4.

**Table 3:** Stratification of the 1-year ISR according to the stented artery and the Mori type.

**Characteristic**	**Number**	**1-year ISR, *N* (%)**	***P*-value**
Location, *N* (%)			0.103
ICA	11	4 (36.4%)	
MCA	37	4 (10.8%)	
BA	44	4(9.1%)	
VA	26	5 (19.2%)	
Mori classification, *N* (%)			0.632
Mori A	11	2 (18.2%)	
Mori B	60	10 (16.7%)	
Mori C	47	5 (10.6%)	

*BA, basilar artery; ICA, internal carotid artery; MCA, middle cerebral artery; VA, vertebral artery; ISR, in-stent restenosis*.

**Table 4:** Comparison of the outcomes between the WEAVE/WOVEN trial and the present study.

	**Outcomes**		**WEAVE/WOVEN**	**Present study**	***P*-value**
72 h after the procedure	Primary	Any stroke or death	4/152, 2.6%	5/130, 3.8%	0.811
	Secondary	Hemorrhagic stroke (without death)	2/152, 1.3%	2/130, 1.5%	1.000
		Ischemic stroke	2/152, 1.3%	2/130, 1.5%	1.000
		Major stroke	1/152, 0.7%	1/130, 0.8%	0.912
		Neurological death	2/152, 1.3%	1/130, 0.8%	1.000
Throughout the follow- up period	Primary	Any stroke or death	11/129, 8.5%	11/125, 8.8%	0.938
	Secondary	1-year stroke or death	11/129, 8.5%	9/125, 7.2%	0.695
		1-year symptomatic ISR	7/102, 6.9%	4/118, 3.4%	0.239
		1-year ISR	18/102, 17.6%	17/118, 14.4%	0.512
		Time to ISR, m	5 (1–11)	9 (5.8–12)	/
		Stroke rate (include death) after 1 month of the procedure	7/129, 5.4%	6/125, 4.8%	0.821
		Major stroke rate after 1 month of the procedure	1/129, 0.8%	3/125, 2.4%	0.592
		Neurological death after 1 month of the procedure	0	1/125, 0.8%	0.233

*ISR, in-stent restenosis*.

## Discussion

The present study suggested the Enterprise stent was technically feasible and safe in treating symptomatic ICAS due to hypoperfusion. With adherence to strict patient selection, careful periprocedural management, and experienced operators, the present study showed that the short-term and long-term outcomes of Enterprise stenting for ICAS were acceptable, which were equivalent to that in the WEAVE/WOVEN trial.

The inclusion criteria of the present study were similar to those of the WEAVE/ WOVEN trial in the degree of stenosis, patient age, and time to stenting after the last onset. However, there were several differences in baseline data between the present study and the WEAVE/ WOVEN trial. There were more male patients (66.2 vs. 53.3%, *P* = 0.028) and a lower prevalence of hypertension, hyperlipidemia, and diabetes mellitus (*p* < 0.05) in the present study than that in the WEAVE/ WOVEN trial. The WEAVE/ WOVEN trial enrolled patients with a recurrent stroke in the territory of the same lesion after receiving medical therapy, while 39.2% of the patients only experienced TIA in the present study. Compared with the WEAVE/ WOVEN trial, there was a higher proportion of posterior lesion (58.5 vs. 35%, *p* < 0.001) especially for BA (36.2 vs. 14%, *p* < 0.001), and higher proportion of baseline mRS score of 0 (69.2 vs. 13.2%, *p* < 0.001). Considered the above differences, it should be cautious to interpret the comparison between the present study and the WEAVE/WOVEN trial.

The present study showed a 100% successful recanalization rate of symptomatic ICAS due to hypoperfusion and the mean stenosis degree improved from 82.9 to 15.1%. Compared with the Wingspan or Apollo stent for symptomatic ICAS in previous studies, there was a higher proportion of Mori C lesions successfully treated by the Enterprise stent in our present study (24.1% for Wingspan and 14.5% for Apollo vs. 40% for Enterprise) (12, 26). This may suggest that the Enterprise stent may be better suitable than the Wingspan or Apollo stent for treating Mori C lesions (long or angular lesions with far tortuous access).

The present study showed that the safety of Enterprise stenting for symptomatic ICAS was acceptable. Compared with the WEAVE/ WOVEN trial, the present study showed a higher prevalence of stroke or death (3.8 vs. 2.6%), a comparable rate of hemorrhagic and ischemic stroke (1.5 vs. 1.3%), and a lower occurrence of death (0.8 vs. 1.3%) within 30 days after the procedure. Compared with the SAMMPRIS trial, the stroke or death rate within 30 days after the procedure in the present study was significantly lower (3.8 vs. 5.7% for the medicine arm and 14.7% for the stent arm). Vajda et al. performed a single-center study in Germany that enrolled 189 patients with 209 ICAS treated with the Enterprise stent demonstrated a stroke or death rate of 7.7% within 30 days after the procedure (18). It should be noted that TIA attributable to the target vessel was also recorded as the outcome, which may increase the event rate. Another two studies in China also used the Enterprise stent for symptomatic ICAS treatment, which yielded a successful operation rate of 100%, and the stroke or death rate within 30 days after the procedure was also low (12, 19). However, the sample size of these two studies was small.

This study showed the long-term outcomes of Enterprise stenting for ICAS were also acceptable. The present study, with its large sample size and long-term clinical follow-up period, showed a stroke or death rate of 8.8%, and a 1-year stroke or death rate of 7.2%, which was lower than that of 8.5% in the WOVEN trial and 20% in the SAMMPRIS stent arm. The Kaplan-Meier curve showed the 1- and 2-year stroke or death rate was 6.5 and 8.9%, respectively (Figure 4). Deployment of an Enterprise stent in the treatment of the symptomatic ICAS appears effective with a lower possibility of stroke recurrence.

Compared with the WOVEN trial, the present study showed a lower 1-year ISR rate (14.4 vs. 17.6%) with a 90.8% imaging follow-up rate. A previous study by Vajda et al. showed a higher ISR rate of 24.7% (43/174) during an average follow-up period of 6.9 months after the Enterprise stenting for ICAS (18). However, in Vajda's study, ISR was defined as more than 50% by DSA that is different from the definitions in our study and the WOVEN trial (more than 70%). Other studies reported that the lower ISR with Enterprise than Wingspan may be attributed to the closed-cell design (11, 14, 16, 17) as well as a less vascular intimal injury due to lower radial force (27). Besides, we think that a low ISR rate of the Enterprise stent may be related to the hemodynamic changes after implantation of the closed-cell stent, which results in a more significant change in the shape of the artery as compared with an open-cell stent.

Our present study showed there was no significant difference in the ISR rate between the three Mori types (Table 3). Previous studies of Apollo stenting for ICAS suggested that the longer the lesion, the higher the ISR rate (26, 28, 29). This may suggest the ISR rate would not affect by lesion length when Enterprise stenting for symptomatic ICAS. The Enterprise stent may be more suitable for treating longer ICAS than Apollo stent, due to the lower rate of ISR.

However, this study has clear limitations. First, this was a retrospective review of uncontrolled clinical data in a single center, so selection bias may exist, and multicenter studies were needed to support our findings and conclusions. Second, although the outcome definition and part of the ascertainment methodology were the same as in the WEAVE/WOVEN trial, there were also some differences in lesion characteristics, ethnicity, and qualifying ischemic events before enrollment, and the findings in the present study thus need to be rephrased and interpreted with caution.

## Conclusion

This study found that the off-label use of Enterprise stent for symptomatic ICAS had a low rate of periprocedural and long-term stroke and neurological death under careful patient selection, stenting performed by experienced neurointerventionalists, and strict periprocedural management. Whether the Enterprise stent is superior or inferior to Wingspan stent system or other intracranial stents in preventing stroke recurrence due to ICAS needs to be confirmed or refuted in future studies.

## Data Availability Statement

The original contributions presented in the study are included in the article/supplementary material, further inquiries can be directed to the corresponding author/s.

## Ethics Statement

The studies involving human participants were reviewed and approved by the Institutional Review Board of Beijing Tiantan Hospital, Capital Medical University, Beijing, China. The patients/participants provided their written informed consent to participate in this study. Written informed consent was obtained from the individual(s) for the publication of any potentially identifiable images or data included in this article.

## Author Contributions

NM and ZM had full access to all of the data in the study, take responsibility for the integrity of the data, the accuracy of the data analysis, and critical revision of the manuscript for important intellectual content. YW, DM, FG, XL, NM, and ZM: study concept and design. RC, LY, KK, MY, and YY: acquisition of clinical data. RC, NM, and ZM: analysis and interpretation of data. RC: drafting of the manuscript. RC and NM: statistical analysis. All authors contributed to the article and approved the submitted version.

## Conflict of Interest

The authors declare that the research was conducted in the absence of any commercial or financial relationships that could be construed as a potential conflict of interest.
